# Influence of Nisin Grafting on the Antibacterial Efficacy of AMP Self-Assembled Monolayers (SAMs)

**DOI:** 10.3390/molecules29225417

**Published:** 2024-11-17

**Authors:** Chloé Richet, Adeline Marguier, Audrey Bertin, Thérèse Leblois, Vincent Humblot

**Affiliations:** Department of Micro Nano Sciences and Systems (MN2S), Université Franche-Comté, UMR 6174 CNRS, FEMTO-ST Institute, F-25000 Besançon, France; chloe.richet@femto-st.fr (C.R.); marguier.adeline@gmail.com (A.M.); audrey25bertin@laposte.net (A.B.); therese.leblois@femto-st.fr (T.L.)

**Keywords:** Nisin coating, antimicrobial peptide, bactericidal effect, antiadhesive properties

## Abstract

The use of antimicrobial peptides (AMPs) covalently grafted on surfaces has been recognized in recent years as a promising strategy to fight against biofilm formation. However, after grafting, the understanding of AMP–bacteria interactions is still debated in the literature. In this study, Nisin, a cyclic AMP, was grafted onto gold surfaces via an indirect grafting on acidic thiol self-assembled monolayers using succinimide linkers. The physical and chemical properties of these SAMs were then finely characterized by XPS and FT-IR to confirm the covalent grafting of Nisin. The antiadhesion and bactericidal effects were then studied for *Escherichia coli* ATCC25922, *Staphylococcus aureus* ATCC 25923, and *Listeria ivanovii* Li4(pVS2) by a posteriori analysis of the culture supernatants (i.e., indirect technique) and ex situ by optical microscopy following crystal violet staining (i.e., direct technique). Statistical analysis reveals that the Nisin coating has bactericidal and antiadhesive properties towards Gram-positive bacteria, while no significant results were obtained for Gram-negative bacteria.

## 1. Introduction

The contamination of surfaces, installations, and equipment by the development of bacterial micro-organisms in the food and health fields (nosocomial infections, discharges, food poisoning), but also cosmetics, pharmaceuticals, and industrial sectors (corrosion of ship hulls, corrosion of water pipes) constitutes a real public health problem [1,2,3].

Described by Donlan and Costerson in 2002 [4], these bacteria communities can organize themselves into groups within self-produced matrices that adhere to each other, and anchor themselves to numerous supports, forming a biofilm. The surfaces on which biofilms form are diverse in nature: natural or organic (rock, skin, internal organs, leaves or roots), industrial (ship hulls, pipes), and medical (protheses, implants, catheters) [5,6]. Biofilms are ubiquitously present and are reservoirs for pathogens that may cause many infections; because of their complex structures and strong attachment to surfaces, their control can be complicated by their resistance to conventional treatments such as germicides and antibiotics [7,8,9].

Several strategies have been proposed to create sterile surfaces to combat bacterial contamination and biofilm growth, by adding antiadhesive, biocidal, or antibacterial compounds via chemical grafting, impregnation, or physical trapping [10,11]. These include quaternary ammonium derivatives [12] or phenolic derivatives [13], antibiotics [14], or heavy metals such as silver or tin, in the form of coatings, dressings, or nanoparticles [15,16,17]. Although a promising strategy, the use of such compounds is compromised by their potential toxicity to humans and to the environment. Moreover, in the case of biocidal compounds and antibiotics, bacteria become resistant after some time of use, and even led to bad reactions in patient bodies [3,18]. This shows the urgent need to find an alternative for antibiotics, through the development of antibacterial molecules and compounds with high efficiency and low toxicity, and that do not induce bacterial resistance.

An alternative to antibiotics is the use of antimicrobial peptides (AMPs), that are naturally produced by plants, mammals and microorganisms, enabling them to defend themselves against bacterial infections [19,20,21]. To date, the Antibacterial Peptide Database (APD, [22]) lists more than 4000 AMPs, which have different properties. These peptides are small, positively charged molecules of 12 to 100 amino acids, naturally present in many organisms, and are produced in areas of infection and inflammation as the infected host’s first immune barrier [23,24]. Although they have similar physical properties, AMPs differ in their secondary structures, which give them different biological properties: antibacterial, antifungal, antiparasitic, and antiviral properties. Recent studies have led to the isolation and characterization of thousands of AMPs in order to determine their biological activities and modes of action [24,25]. Despite their promise, natural AMPs have medium-range production costs and a narrower range of applications than synthetic AMPs. However, one natural peptide is an exception: Nisin, a bacteriocin produced naturally by the fermentation of *Lactococcus lactis bacteria* [26].

Nisin is one of the so-called “lantibiotics”, a family of peptides made up of non-usual amino acids. It is 34-amino-acid-residues long and has several configurations, the best known and the most active being Nisin A and Nisin Z [26,27,28,29]. Nisin Z was discovered in 1991 and is one of the natural variants of Nisin A that differs in that its residue in position 27 is Asparagine instead of Histidine [30]. While Nisin has been shown to be effective against Gram-positive bacteria such as *Salmonella, Pseudomnas*, *Staphylococcus*, and *Listeria* [31,32,33], its effect on Gram-negative bacteria, such as *E. coli*, remains highly debated [34]. The insensitivity of Gram-negative bacteria to Nisin may be explained by the impermeability of the outer membrane of this type of bacteria, which is normally anionic due to the acid groups present on its surface, the latter annihilating the peptide’s effect [35]. In addition, some authors have studied several parameters that clearly affect the efficiency towards Gram-negative membranes such as the structure characteristics related with bactericidal actions, including peptides’ constituents, molecular lengths, molecular charges, and secondary structures. In particular, the absence of an amphipathic design in the structure is responsible for low bactericidal activity [36]. This last point is clearly crucial in the mode of action of the peptide, namely its pore forming and inhibition of the cell wall’s synthesis [37].

Early studies focused on unpurified Nisin, which had no antibacterial effect against Gram-negative bacteria. The Minimum Inhibitory Concentration (MIC) values of Nisin against *S. aureus* (Gram-positive) and *E. coli* (Gram-negative) were investigated by Kuwano, who again showed that unpurified Nisin has no bactericidal effect on *E. coli* (MIC > 75 µM) [34]. However, he also studied the MIC values for purified Nisin on the same two bacteria. The work showed that the MIC for *E. coli* (600 nM) was eight times higher than the MIC for *S. aureus* (75 nM). The antibacterial activity of Nisin against Gram-negative bacteria is still controversial, and a recent study showed some antibacterial activity of Nisin against 300 strains of Gram-negative bacteria, with a high sensitivity for *Helicobacter, Xanthomonas*, *C. freundii*, but not for *E. coli* [38,39].

The wide range of Gram-positive bacteria that can be treated with Nisin (alone or in combination with other molecules) makes it the most widely used antimicrobial peptide in the food industry, especially in food packaging, to limit bacterial growth or even eliminate contamination [40,41]. McAuliffe has widely studied the mechanism of action of Nisin, showing that the peptide targets lipids II on the surface of the bacteria, before adsorbing and starting to destabilize their cytoplasmic membrane structure, at very low concentrations [42]. It has also been reported that Nisin is effective against the adhesion of some Gram-positive bacteria such as *Salmonella*, *Pseudomonas*, or *Listeria ivanovii* on polymer-coated stainless steel [43,44], and against *Listeria monocytogenes* in many conditions [31].

Numerous strategies for grafting these efficient molecules onto surfaces have been developed. Among them, we have (i) polymer brushes used by Glinel [45] or Hadjesfandiari [46], (ii) multilayer polyelectrolyte films functionalized by the insertion of defensin [11], and (iii) chitosan film onto which the Dhvar5 peptide is covalently grafted onto titanium and gold surfaces [47]. Although these methods have interesting properties, they do not always prevent the formation and growth of biofilms on the surface [48]. This fact leads to the development of a strategy that first prevents bacterial adhesion on the surface and kills bacteria with one or more biocidal agents. Humblot et al. in 2009, used one of the widely used strategies to graft Magnanin I, creating self-assembled monolayers (SAMs) on which the biological active compounds can be immobilized [49]. Indeed, molecules used to create SAMs are usually long and form a dense layer on the surface, onto which antibacterial molecules can be grafted, thus preventing bacterial adhesion and providing a biocidal effect to the surface [50].

**Figure 1:**
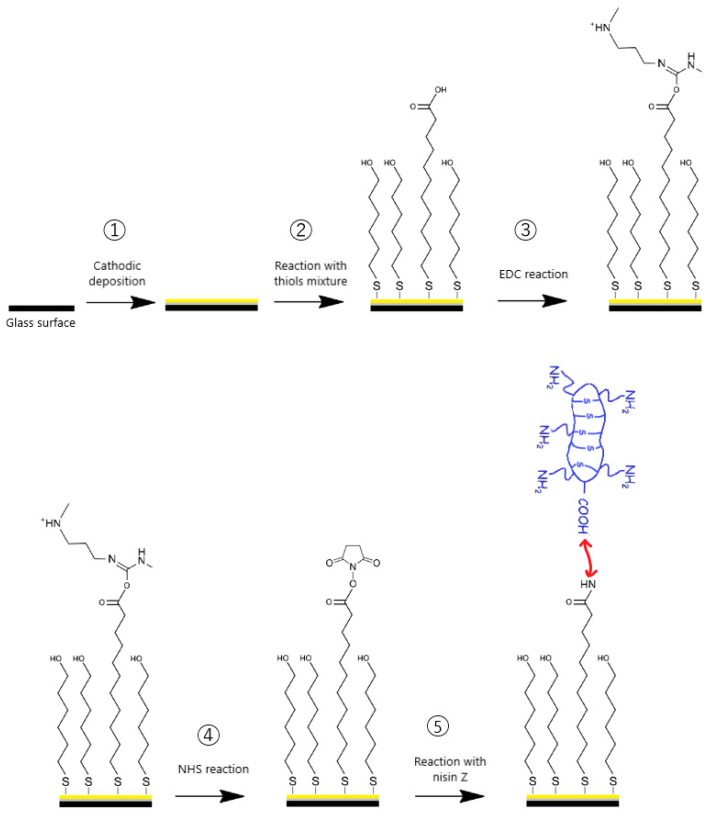
Nisin grafting strategies on COOH thiol self-assemblies.

Some studies have shown the antiadhesive and antibacterial effect of Nisin against algae and *Listeria monocytogenes* [44,51]. In the present paper, we studied antibacterial and antiadhesive properties of Nisin Z against *Escherichia coli* (Ec), *Staphylococcus aureus* (Sa), and *Listeria ivanovii* (Li). The strategy used for the grafting of Nisin on gold surfaces is the fabrication of mixed thiol (25:75, MUA:C_6_OH) SAMs, which involves grafting of Nisin on to acid thiols activated via a succinimide linker. Covalent binding of Nisin was performed by the conversion of carboxylic terminal groups of MUA into esters, thanks to the reaction between N-hydroxysuccinimide (NHS) and carbodiimide (EDC), followed by the reaction between esters and one of four NH_2_ of Lysin residue or an N-ter group (Figure 1).

Physico-chemical characterization has been performed by FT-IR and XPS spectroscopies to confirm the grafting of Nisin. Then, antiadhesion and antibacterial effects were evaluated for the three bacteria, *Echerichia coli* ATCC 25922, *Listeria ivanovii* Li4 (pVS2), and *Staphylococcus aureus* ATCC 25923, by optical microscopy and the culture of supernatants. A statistical analysis of the results is performed to determine if Nisin has antiadhesive or antibacterial effects once grafted on the surface.

## 2. Results and Discussion

### 2.1. Grafting Characterizations

**Figure 2:**
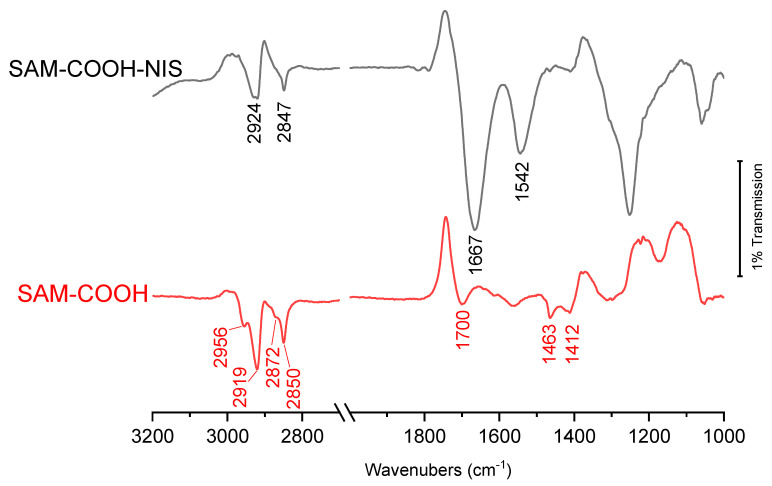
FT-IR ATR spectra of Nisin grafted on SAM-COOH thiol.

Figure 2 presents the FT-IR ATR data obtained for COOH thiol self-assembled monolayers (SAM-COOH) and after the grafting of Nisin (SAM-COOH-NIS) on Au surfaces. First, the SAM-COOH spectrum exhibits feature at ~1700 and in the 1412–1463 cm^−1^ region assigned to v_C=O_, mainly [52,53,54,55], and to several modes of vibrations of CH_2_ moieties from the backbone of the molecule. It is important to note that both spectra exhibit a strong signal for sym and asym stretching of CH_2_ at 2919–2924 and 2850–2847 cm^−1^. In addition, the SAM-COOH spectrum exhibits traces of sym and asym stretching of CH_3_ due to the purity of the thiols being around 95%. Nisin was thus grafted on SAM-COOH via an amino group on activated SAM-COOH (Figure 1). In the spectrum at the top of Figure 2, one can see the appearance of Amide I and II bands at 1667 and 1542 cm^−1^, respectively, showing the successful grafting of Nisin.

**Figure 3:**
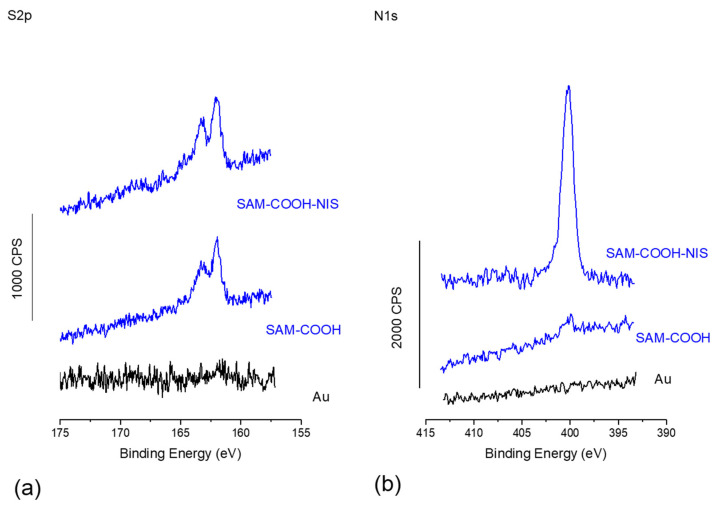
(**a**) XPS S2p high-resolution (HR) region and (**b**) N1s HR region for Au, SAM-COOH, and SAM-COOH-NIS.

In order to confirm this point and to obtain quantitative data on these SAMs, XPS analyses were performed on the two surfaces, and the data are presented in Figure 3. First, Figure 3a shows the S2p high-resolution region, where the S2p_1/2_ and S2p_3/2_ doublets can be observed confirming the successful functionalization of both SAM-COOH. After Nisin grafting, these signals are enlarged due to the contribution of five disulfuric bridges in the Nisin molecules [30], again confirming the successful grafting of the peptide. Atomic percentages are presented on Table 1. The atomic percentage of S2p does not vary much as the S2p signal coming from the thiol anchoring moieties are attenuated by the Nisin molecule, together with the contribution from the five S atoms of the peptide.

When looking at Figure 3b, one can see even more clearly the successful grafting of the NIS molecule with the presence of an intense broad peak in the 400 eV region, suggesting the presence of protonated and non-protonated amino groups. Again, the Nisin grafting is confirmed when looking at the atomic percentage of Table 1 for the N1s region, with a huge increase of around 800% of the N1s atomic percentage. Finally, the decrease in the Au4f signal also confirms the increase in the organic layer on top of the SAM-COOH, hence the grafting of Nisin.

From XPS quantitative data presented on Table 1, it is possible to estimate the equivalent thicknesses of the different layers created on the gold surfaces and the associated surfaces’ coverage using Equations (1) and (2), described in the Section 3. Thus, for the SAM-COOH, the thiol surface coverage is calculated at 5.6 thiols/nm^2^, from data from Table 1 and Equations (1) and (2). This surface coverage is relatively close to the optimal theoretical thiol coverage of 6–8 thiols/nm^2^. When looking at the coverage obtained once Nisin has been grafted on acidic SAM, we obtain an equivalent coverage of 0.55 nisin/nm^2^. These values are quite common when looking at equivalently massed and sized molecules grafted on surfaces, with for instance 1.3–1.9 temporine/nm^2^ on titanium (13 AA for Temporin vs. 34 AA for Nisin) [56].

### 2.2. Microbiological Tests

Several tests were carried out to attest to the behavior of our coatings towards several bacterial strains. Bacterial adhesion as well as killing experiments were performed and killing efficiency will be discussed as a function of the reference chosen for these calculations. They were carried out following an indirect route, based on the recovery of adhered bacteria and viable cell culture counting on agar plates. The first test was carried out in order to evaluate the adhesive properties while the second one enabled the calculation of the bactericidal activity of the grafted Nisin coating.

#### 2.2.1. In Vitro Tests

First tests in solution were performed in order to evaluate the potential activities of Nisin in solution, and Table 2 presents the Minimal Inhibition Concentration (MIC) and Minimal Bactericidal Concentration (MBC) obtained for these three bacteria.

First, we can observe that Nisin has no antibacterial activity or a potential very low one outside of the range of our experimental concentration for determining the MIC (>20 mg/mL) towards Gram-negative *E. coli* bacteria, as expected from data from the literature [57] since the outer membrane (OM) of Gram-negative bacteria acts as a impermeability barrier for the cell and prevents Nisin from reaching the cytoplasmic membrane [42].

Charest [38] studied Nisin activity against 17 genera of Gram-negative bacteria and published a range of MIC for each. The different strains were then classified in four groups: high sensitivity, moderate sensitivity, low sensitivity, and no detected sensitivity. Fifty-one strains of *E. coli* were tested and 50 of them showed an MIC mean value around 171.81 μg/mL, which is surprisingly low compared to what we obtain [38]. Other Nisin-sensitive genera include *Xanthomonas, Erwinia*, and *Helicobacter* that are sensitive to Nisin for mean concentrations between 5 and 130 µg/mL. Nisin’s bactericidal effect against Gram-negative is still debated currently, especially the mechanism of action against *E. coli*. Therefore, Nisin cannot been considered as an antimicrobial peptide against *E. coli*. The tendency of Nisin to be not bactericidal towards Gram-negative bacteria could be a problem as Nisin is one of the rare AMPs to be FDA approved.

Around 84% of the natural AMPs of the antimicrobial peptide database (APD3) have been registered as being antibacterial, having Gram-positive activity, anti-Gram-negative effects, or effects against both types of bacteria [22]. Unfortunately, these promising molecules are still being studied in trials or in vitro investigations to show their effect against Gram-negative bacteria. Only one of the seven peptides approved by the FDA has an effect against Gram-negative bacteria, and two are still under investigation [58,59]. Oncocin and its derivates seem to be efficient against some Gram-negative bacteria such as *E. coli* and *M. luteus*. Depending on the sequence of the peptide used, derived from *Oncopeltus,* 13 peptides have been discovered [60]. For example, peptide 4 is efficient against *E. coli* and *M. luteus* with an MIC of about 8 to 128 of µg/mL, peptides 10 and 11 are efficient against both those cited and also against *P. aeruginosa*, *K pneumoniae*, and *E. cloacae* strains, showing a large broad spectrum of MICs from 0.25 to 8 µg/mL [60].

However, when turning to Gram-positive bacteria, Nisin shows much better antibacterial activity in the range of 156 µg/mL towards *Listeria* and 312 µg/mL for *S. aureus*, which are close values to those that can be found in the literature [33].

Tyrocidin has been found to be efficient against both Gram-positive (Sa) but not Gram-negative (Ec) bacteria but it also exhibited a toxicity for human blood cells [61]. Another small peptide, indolicilin, composed of only 13 amino acids, is efficient against both Gram-positive and Gram-negative bacteria [62]. In the work of Falla et al. [63], MIC values are relatively low against, notably, the wild type *E. coli* (4 to 16 µg/mL), the defensin supersensitive *S. typhimurium* (8 to 64 µg/mL), and *Staphyloccocus spp* (4 and 8 µg/mL). Two other peptides were also tested and the MICs that are shown are also low against the previous cited strains, from 0.25 to 32 µg/mL for Gentamicin and from 0.25 to 64 µg/mL for Polymixin B. King and Phillips [64] have tested vancomycin and penicillin against a large range of Gram-positive strains of *Staphylococci*, including those that are methicillin-susceptible and methicillin-resistant. Daptomycin was efficient against all tested strains, with an MIC between 0.03 and 1mg/mL, depending on the method used.

#### 2.2.2. Surfaces Tests

First experiments carried out were aimed at the influence of the coatings towards bacterial adhesion. In fact, it is known that, depending on the surface charges and some other parameters, such as surface free energy, the bacterial adhesion can be reduced, which can be a first effect on the global efficiency of the coatings.

**Figure 4:**
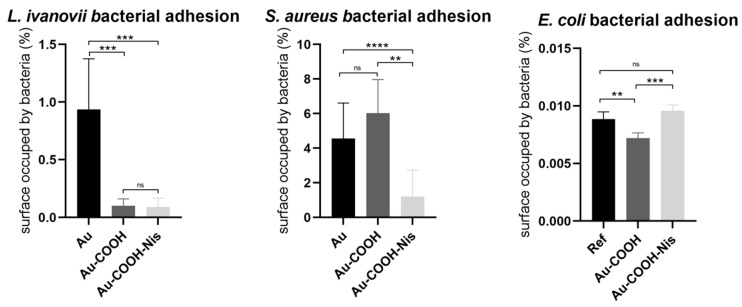
Bacterial adhesion on three surfaces: bare Au, SAM-COOH, and SAM-COOH-NIS. Bacteria tested were *Listeria ivanovii* (Li), *Staphylococcus aureus* (Sa), and *Escherichia coli* (Ec). Adhesion incubations were performed at 30 °C (Ec) and 37 °C (Li and Sa) in PBS at an initial concentration of 10^6^ CFU/mL. (ns: non-significant; **: *p* < 0.01; ***: *p* < 0.005; ****: *p* < 0.001).

Figure 4 shows the results obtained after 3 h of incubation of several bacteria (*Listeria ivanovii* (Li), *Staphylococcus aureus* (Sa), and *Escherichia coli* (Ec)) on three different surfaces: bare gold (absolute reference surface), SAM-COOH (functionalized reference surface), and finally SAM-COOH-NIS.

Looking first at the Gram-positive bacteria Li and Sa, the results are quite different when compared to the bare Au surface. Indeed, for Li, a strong decrease in the adherence by one order of magnitude is observed for both functionalized surfaces. On the contrary, for Sa, the thiol layer increases the adherence by 50% while SAM-COOH-NIS reduces the bacterial adhesion by a factor of three the bacterial adhesion. These different behaviors could be explained by the intrinsic properties of the bacteria themselves, with *L. ivanovii* being a motile bacterium [65] while *S. aureus* does not have the same mobility properties [66].

From another point of view, when looking specifically at the antibacterial coating on the adhesion, the Nisin coating does not influence much bacterial adhesion for *L. ivanovii* while it clearly has an influence for *S. aureus* with an adhesion decrease of almost a factor of five when compared to the SAM-COOH surface. Nevertheless, the adhesion of Li is already drastically reduced compared to bare gold, thus the difference between SAM-COOH and SAM-COOH-NIS, even if it is neglectable, could still show some interest compared to a totally bare surface. Finally, in the case of Gram-negative bacteria, i.e., *E. coli*, there are no significant differences for all three considered surfaces.

Killing-by-contact experiments were carried out in order to evaluate the bactericidal efficiency of covalently grafted Nisin towards the same three bacterial strains on surfaces. Here, it is important to note that incubation was performed in PBS after washing the inoculum by centrifugation to be sure that no growing media was left, and that no increase in CFU due to bacteria proliferation could be possible.

**Figure 5:**
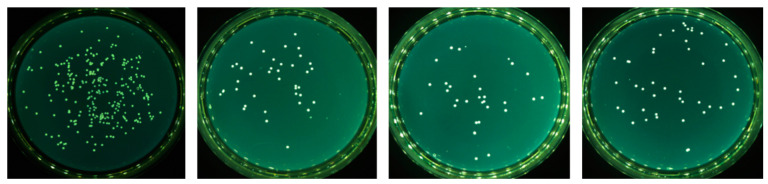
Examples of CFU counting on agar plate for Sa, from **left** to **right**: inoculum, Au, SAM-COOH, and SAM-COOH-NIS.

**Figure 6:**
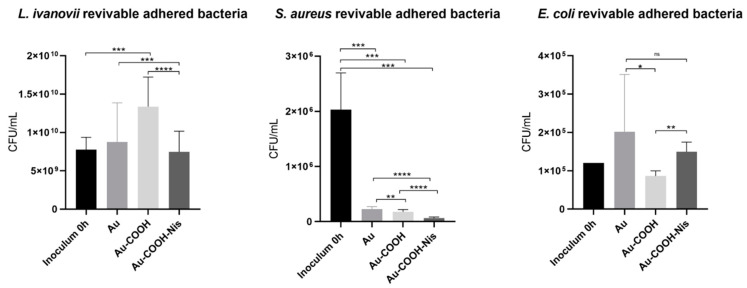
Revivable adhered bacteria recorded on three surfaces: bare Au, SAM-COOH, and SAM-COOH-NIS. Bacteria tested were *Listeria ivanovii* (Li), *Staphylococcus aureus* (Sa), and *Escherichia coli* (Ec). Adhesion incubations were performed at 30 °C (Ec) and 37 °C (Li and Sa) in PBS; initial CFU/mL concentrations are given as inoculum value for each separate experiment. (ns: non-significant; *: *p* < 0.05; **: *p* < 0.01; ***: *p* < 0.005; ****: *p* < 0.001).

Figure 5 presents examples of CFU/mL of revivable adhered bacteria recovered from the different surfaces and counted after overnight incubation on agar gelose, where every single dot represents a bacterial CFU and Figure 6 presents the data obtained for all three bacterial strains.

In addition, Table 3 presents the killing efficiency in % with respect to different references following the following formula (Equation (3)):

With the ref being the initial bacterial inoculum, the bare Au surface, or the SAM-COOH surface.

Indeed, depending on the literature, the field of research, or simply the research group, the reference used for calculating the killing efficiency is very variable. Therefore, here, for treating the raw data (i.e., before any correlation with bacterial adhesion), we have decided to calculate the % of killing with respect to three different references: the bacterial inoculum, the raw surface, and the functionalized surface at the step just before Nisin grafting. Figure 6 presents the data obtained for revivable bacteria counted for each surface after inoculation of bacterial suspension.

First, when looking at data from Table 3 and Figure 6, it is striking to see that depending on the reference used, the result for the killing efficiency is very different, and with no logical scheme when turning from one bacterium to another bacterial strain.

When looking first at the functionalized surface at the step just before Nisin, i.e., the % killing/SAM-COOH, this seems to be the best reference to attest to the efficiency of the grafted Nisin peptide. As expected from the literature, Nisin seems to have no effect towards Gram-negative *E. coli*, with a negative killing efficiency. On the contrary, for Gram-positive strains, a moderate killing efficiency is obtained with around 48% and 64%, respectively, for *L. ivanovii* and *S. aureus*. The difference is not significative and follows what was found in solution for the MIC/MBC experiments (Table 2), with similar values. In addition, previous studies have shown that other AMPs (namely Magainine I and Gramicidine) grafted on gold COOH SAMs exhibit a killing efficiency that is similar for both *L. ivanovii* and *S. aureus* with, respectively, 70 and 80 % for Magainine [49] and 70% and 65% for Gramicidine [67].

When looking at the bare initial surface used as a reference, the killing efficiencies observed are within the same tendency, with a higher activity towards *S. aureus* than towards *L. ivanovii* with still better activity for coqui than for bacillus. Surprisingly, when using the bare surface as a reference, the Nisin coating exhibits a very small activity towards *E. coli*, going thus against the in vitro MIC and MBC experiments.

Finally, using the inoculum as a reference for calculating the killing efficiency of grafted Nisin, the results are totally different with almost no activity towards *Listeria* (1.2%), a much higher amount of activity towards *S. aureus* (~97%), and still a negative activity towards *E. coli*.

It is thus quite difficult to make a final choice on how to present the killing efficiency data, as many debates are present in the literature and no researcher uses the same reference. However, an international normae was published stating that in the presence of an untreated control surface, if the killing efficiency between the inoculum and the untreated surface differs by more than 15%, the latest case should be preferred [68]. In our case, clearly the 15% difference is reached; therefore, for the rest of our analysis, only the untreated surface will be used for calculating any killing efficiency.

Finally, another way of combining all the data obtained previously could be to make a correction to the adhered revivable bacteria by the adhesion factor calculated from Figure 4 (adhesion factor obtained by CFU normalization against Au surface. Corrected CFU are raw CFU × Adhesion factor). In fact, if a surface shows little adherence, the overall bactericidal efficacy will be increase with respect to the surface colonization by bacteria, thus Table 4 shows the corrected values for the killing efficiencies. The tendency is still the same, with the Nisin coating being more efficient towards *S. aureus* than towards *L. ivanovii.* However, due to the low adhesion of Gram-positive bacteria (specially for *S. aureus*) on the Nisin coating, the killing values are increased, reaching up to 66% of killing for Li and 92% for Sa.

Using a corrective factor linked to the bacterial adhesion enables us to obtain a general view of the potential efficacy of the Nisin coating to reduce surface colonization and hence biofilm formation.

Based on the results obtained with the coating of Nisin on gold surfaces via an acidic thiol self-assembled monolayer, few points of improvements could be explored.

It appears clear that the purity of the antimicrobial peptide is of extreme importance with regard to the antibacterial activities of free peptides or immobilized peptides (physically or covalently) [34]. Therefore, some ongoing work is performed in our research group to purify Nisin using High-Performance Liquid Chromatography (HPLC) or Cation Exchange chromatography (IEC). With the same idea, the optimization of nisin production (followed by high purification) can also help to increase the bacterial activity of home-made produced Nisin, using, for instance, a specific strain of L. lactis, which is less commonly used in industry [69].

Finally, the orientation of the AMP with respect to the surface could also be crucial for bactericidal activities. Indeed, using the classical surface chemistry of acidic thiols for the grafting of AMPs on surfaces is the purpose of this work. However, as Nisin possesses five possible amino anchoring groups, the orientation of the peptide will be random and heterogenous through the whole surface. On the other hand, using amino thiol could solve this problem as Nisin possesses only one COOH group in its lateral position, hence using the reverse route (activation of the COOH of the Nisin and grafting directly on the amino SAM) will result in the same orientation of the peptide on the overall surface. We have shown previously that this orientation can strongly influence the bactericidal activity of grafted AMPs [56,70].

## 3. Materials and Methods

### 3.1. Grafting Strategies

#### Chemical and Surface Preparation

Nisin (Ile-Dhb-Ala-Ile-Dha-Leu-Ala-Aba-Pro-Gly-Ala-Lys-Aba-Gly-Ala-Leu-Met-Gly-Ala-Asn-Met-Lys-Aba-Ala-Aba-Ala-His-Ala-Ser-Ile-His-Val-Dha-Lys), 6-mercaptohexanol (C_6_OH), 11-mercaptoundecanoïc acid (MUA), 1-(3-dimethylaminopropyl)-*N*-ethylcarbodiimide hydrochloride (EDC), and N-hydroxysuccinimide (NHS) were purchased from Sigma Aldrich (Saint-Quentin Fallavier, France). Reagents were used without any further purification and experiments were carried out at room temperature if not specified otherwise. The purity of Nisin is not given, no attempt to purify it was performed, despite the fact that it is more likely that it is a mixture of Nisin A and Nisin Z.

The surfaces were constituted of glass substrates (11 mm × 11 mm), modified by cathodic deposition with Plassys MP 700 in a Mimento Renatech Technology Center clean room. Glass substrates were first activated by argon plasma then coated successively with a 5 nm thick layer of chromium and a 200 nm thick layer of gold. Gold surfaces were then cleaned in a bath of absolute ethanol for 5 min before the adsorption of thiols.

Cleaned gold surfaces were immersed overnight at room temperature in a binary mixture of 1 mM (25/75) of MUA (0.25 mM) and C_6_OH (0.75 mM) in absolute ethanol, under magnetic stirring, in order to insure an optimal homogeneity of the thiol layer. Surfaces were then rinsed in ethanol and MilliQ water baths for 5 min and dried under a flow of dry nitrogen.

The substrates were treated with a solution of NHS (200 mM) and EDC (50 mM) in ultrapure water for 60 min, rinsed in MilliQ water, and dried under a flow of dry nitrogen.

Immobilization of Nisin (NIS, 50 mg/L in PBS) on gold surfaces was carried out by depositing a 150 µL drop of NIS/PBS solution on the Au-modified substrates at room temperature for 2 h. After the immobilization step, the surfaces were vigorously rinsed in PBS then MilliQ water with agitation, dried under a flow of dry nitrogen and stocked at 4 °C.

For each step of functionalization, one series of samples was characterized by Fourier-transform infrared spectroscopy (FT-IR) and by X-ray photoelectron spectroscopy (XPS).

### 3.2. Characterization Techniques

#### 3.2.1. ATR-FTIR

The gold samples were placed in the external beam of a Diamond ATR FT-IR instrument (Perkin Elmer Spectrum Two, Waltham, MA, USA) and the reflected light was focused on a DTGS (Deuterated TriGlycine Sulfate) wide band detector. Spectra were acquired at an 8 cm^−1^ resolution with 1 min of co-addition (around 60 spectra), thus ratioed towards a background collected in air. Baseline correction was applied with no further correction. Spectra were plotted as % transmissions.

#### 3.2.2. XPS Analyses

XPS analyses were performed using an Scienta Omicron (Uppsala, Sweden) Argus X-ray photoelectron spectrometer, equipped with a monochromated AlKα radiation source (hν = 1486.6 eV), and 150 W electron beam power. The emission of photoelectrons from the sample was analyzed at a takeoff angle of 45° for Omicron Argus X-ray under ultra-high vacuum conditions (≤ 10^−9^ mbar). Spectra were carried out with 100 eV of pass energy for the survey scan and 20 eV of pass energy for the C1s, O1s, N1s, S2p, and Au4f regions. Binding energies were calibrated against the Au4f binding energy at 84.0 eV and element peak intensities were corrected by Scofield factors [71], the spectra were fitted using the Casa XPS v.2.3.13 Software (Casa Software Ltd., Teignmouth, Devon, UK) and applying a Gaussian/Lorentzian ratio, G/L, equal to 70/30.

Equivalent thickness (*d*) and surface density (*n*) were calculated using the following equations, respectively:

(1) $$\frac{I_{S2p}}{I_{Au4f}}=\frac{\rho_{SAM+Nis}M_{Au}\sigma_{S2p}T_{S2p}\lambda_{S2p}^{SAM+Nis}(1-exp(\frac{-d}{\lambda_{S2p}^{SAM+NIS}sin(\theta )})}{\rho_{Au}M_{COOH+NIS}\sigma_{Au4f}T_{Au4f}\lambda_{Au4f}^{Au4f}(\frac{-d}{\lambda_{Au4f}^{SAM+NIS}sin(\theta )})}$$

where *θ* is the photoelectron collection angle. *T_Au4f_* and *T_S2p_* are the relative sensitivity factors of *Au* and *S*, respectively, provided by the spectrometer manufacturer. The Scofield photoionization cross-sections σ are equal to 14.4 for *Au*4*f* and 1.44 for *S*2*p*. $\lambda_{x}^{y}$ is the inelastic mean free path of electrons x in the matrix y. These were calculated using the Quases program (QUASES-IMFP-TPP2M Ver.3.0) based on the TPP2M formula. *ρ_SAM_*, *ρ_SAM+Nis_*, and *ρ_Au_* are the densities of SAM, SAM + Nis, and Au, respectively. *MSAM*, *MSAM+Nis*, and *MAu* are the molecular weights of SAM or SAM + Nis and Au, respectively, and finally, *I_x_* is the raw intensity of element x.

(2) $$n_{Nis}(nis.{nm}^{-2})=\frac{d_{Nis}\rho_{Nis}N_{A}}{M_{Nis}}$$

where *d_Nis_* is the Nisin equivalent thickness, *ρ_Nis_* is the density of Nisin, *M_Nis_* is the molecular weight of Nisin, and *N_A_* is Avogadro’s number.

%Killing = 100 × ((bacteria CFU ref − bacteria CFU SAM-COOH-NIS)/bacteria CFU ref)(3)

### 3.3. Microbiological Tests

#### 3.3.1. Surface Activity Tests

##### Bacteria Strains, Media, and Culture Conditions

The bacterial strains used in this work are *Listeria ivanovii* Li4pVS2, *Escherichia coli* ATCC25922, and *Staphylococcus aureus* ATCC25923. All strains were stocked at −80 °C in glycerol aliquot. The inoculum was prepared by first growing a solid culture on biological agar (15 mg/L) + LB (20 mg/L) for *E. coli*, biological agar (15 mg/L) + BHI (20 mg/L) for *L. ivanovii*, and biological agar (15 mg/L) + MH (20 mg/L) for *S. aureus.* Petri dishes were incubated overnight at 30 °C for *E. coli* and 37°C for *L. ivanovii* and *S. aureus*. Thus, liquid cultures were carried out by recovering 1 colony from the solid growth Petri dish and inoculated in 10 mL of associated media at 20, 15, and 20 mg/L for BHI, LB, and MH and cultured overnight at 30 °C (*E. coli)* or 37 °C (*L. ivanovii, S. aureus)* under 90 rpm of agitation.

#### 3.3.2. Contact Killing

Exponentially growing bacteria in media were harvested by centrifugation (5000× *g*, 5 min, 25 °C), washed twice with PBS, and suspended in PBS to obtain a concentration of 10^9^ CFU/mL. Three solutions were prepared for each strain by dilution at 10^8^ CFU/mL, 10^7^ CFU/mL, and 10^6^ CFU/mL in PBS. A measure of 20 μL of the bacterial suspensions was spread manually onto 89 mm Petri dishes filled with agar + suitable media (LB for *E. coli*, MH for *S. aureus*, BHI for *L. ivanovii).* Thus, functionalized gold slides were deposited face-down on the freshly incubated Petri dishes, avoiding the creation of air bubbles. After overnight incubation at 30 °C (*E. coli*) and 37 °C (*S. aureus* and *L. ivanovii*), dishes’ pictures were recorded using an Interscience colony counter Scan 300.

#### 3.3.3. Minimal Inhibition Concentration (MIC)

MIC values toward *bacteria* were determined using the 2-fold dilution method. Experiments were performed in 96-well microplates in triplicate in culture media (LB for *E. coli*, MH for *S. aureus*, and BHI for *L. ivanovii*), with an initial bacterial concentration of approximately 10^6^ CFU/mL. The highest Nisin concentrations prepared were 80 mg/mL, resulting in concentrations in the first well of 20 mg/mL, respectively. After overnight incubation at 30 °C and 37 °C (respectively, for Ec, and Sa and Li), MIC values were determined as the lowest concentration of the compound with no visible bacterial growth. Sterility control (culture broth only), growth control (culture broth with bacteria), and death control (culture broth with bacteria and ethanol: H_2_O *v*/*v* 70:30) were used to assess the quality of each experiment.

#### 3.3.4. Adhesion of Bacteria on Gold Samples

Gold samples were washed successively in 70% ethanol and sterile water then dried in a sterile environment. Surfaces were then immersed in bacteria suspension at 10^7^ CFU/mL in PBS, in a 12-welled plate, and incubated 3 h at 30° C (*E. coli*) and 37 °C (*S. aureus* and *L. ivanovii*) under 90 rpm of agitation. Following incubation, samples were washed 3 times for 5 min with sterile PBS and then dried under a sterile environment.

#### 3.3.5. Optical Microscopy

Bacterial adhesion was measured by analyzing the plates that had undergone the contact tests. After incubation with the bacterial solutions, each sample was washed three times with milli-Q water, then immersed in a homemade 0.5% crystal violet solution in ethanol for 10 min. After staining, the surfaces were rinsed with milli-Q water until the staining disappeared from the well water and then the surfaces were dried. Each surface was observed under a Nikon ECLIPSE LV100ND (Nikon Europe B.V., Amstelveen, The Netherlands) optical microscope at magnifications of ×50 and ×100. Ten photos were taken for each magnification and each sample. The analysis was then carried out using ImageJ software, version 1.54k 15 September 2024.

#### 3.3.6. Statistical Analysis

Graphs and statistical analyses were obtained using GraphPad Prism 8.3.0 software (GraphPad Software Inc., Boston, MA, USA). Mann–Whitney tests were performed to study the significance between conditions directly from raw data, all performed tests are nonparametric tests, unpaired, comparing ranks. The latter was considered statistical when the p-factor *p* < 0.05.

## 4. Conclusions

In this paper, we have successfully covalently grafted Nisin, an antimicrobial peptide of the bacteriocin family, on a mixed acidic thiol self-assembled monolayer, as confirmed by FT-IR and XPS spectroscopies. In addition, XPS data have enabled the average surface coverage of Nisin to be calculated to around 0.6 peptide/nm^2^; this value is within the average coverage usually observed considering the molecular weight and hence the steric hindrance of the AMP Nisin.

In vitro antimicrobial tests have shown that Nisin in solution has no activity towards Gram-negative *E. coli* bacteria, as expected from the literature, while it exhibits middle-range activities towards Gram-positive bacteria with an MIC measured at 156 µg/mL and 312 µg/mL for *L. ivanovii* and *S. aureus*, respectively. Antimicrobial activities were also tested on Nisin-grafted gold surfaces, looking at two parameters: bacterial adhesion and bactericidal efficiency.

Looking first at adhesion, Nisin coatings have no effect on *L. ivanovii*, while the bacterial adhesion decreased by 73% for *S. aureus*. In the case of *E. coli*, the AMP coating shows a small increase in bacterial adhesion.

Antibacterial activity was then measured by counting the number of revivable adhered bacteria on the three kinds of surfaces, the Au reference, the SAM-COOH, and finally the SAM-COOH-NIS. Percentages of killing were calculated using different references according to several methods in the literature: the initial bacterial inoculum, the bare surface, or the non-coated SAM-COOH. Surprisingly enough, the % killing calculated was very different depending on the reference used. For instance, for *S. aureus*, this value goes down from 96.8, to 72.2, to 64.4 %, respectively. While for *L. ivanovii*, the tendency is reversed, with % killing going up from 1.2, to 41.9, to 48.0 %. This debate is not often discussed in the literature and all three references are used depending on the authors of the study, with sometimes very different results from one study to another. In our case, we have chosen to adopt the non-coated surface SAM-COOH as a reference, as recommended by ASTM normae. Using this reference, the Nisin coating is more bactericidal towards *S. aureus* than *L. ivanovii*, and shows no efficiency towards *E. coli*. Finally, a correction of the efficiency was carried out by introducing the adhesion factor in the calculation of the killing, showing final efficiencies for the Nisin coatings of around 66 and 93%, respectively, for *L. ivanovii* and *S. aureus*. Considering the mild activity measured in vitro (MIC determination), the overall surface efficiency of Nisin towards Gram-positive bacteria can be considered as satisfactory. The results also suggest that Nisin coating can have a better activity towards non-motile Gram-positive bacteria with a very low adhesion factor, which was observed for *S. aureus* together with almost 1 log reduction in bacterial viability.

## Figures and Tables

**Table 1:** Atomic percentages obtained by XPS for both SAM-COOH and SAM-COOH-NIS.

	C1s	O1s	N1s	S2p	Au4f
SAM-COOH	59.5	11.4	0.9	2.6	25.6
SAM-COOH-NIS	61.7	13.4	8.1	2.4	14.4

**Table 2:** MIC and MBC of Nisin towards *E. coli*, *S. aureus*, and *L. ivanovii* bacteria.

Nisin	*L. ivanovii*	*S. aureus*	*E. coli*
MIC	156 µg/mL	312 µg/mL	>20 mg/mL
MBC	312 µg/mL	312 µg/mL	>20 mg/mL

**Table 3:** Killing efficiency of Nisin towards three bacterial strains. % are calculated using Equation (3).

	% Killing/Inoculum	%Killing/ref. Surface	% Killing/SAM-COOH
*Listeria ivanovii*	1.2	41.9	48.0
*Staphylococcus aureus*	96.8	72.2	64.4
*Escherichia coli*	<0	23.2	<0

**Table 4:** Killing efficiency of Nisin-coated surfaces, calculated using the non-coated surface as a reference and corrected or not by the adhesion factor.

	% Killing/SAM-COOH	% Killing/SAM-COOH Corrected by Adhesion
*Listeria ivanovii*	48.0	66.4
*Staphylococcus aureus*	64.4	92.6
*Escherichia coli*	<0	<0

## Data Availability

The raw data supporting the conclusions of this article will be made available by the corresponding author on request.
